# Cecal Duplication Cyst Presenting as Acute Intestinal Obstruction in an Infant

**Published:** 2011-03-10

**Authors:** Lubna Ijaz, Muhammad Husnain, Shahid Iqbal Malik, Bilal Mirza

**Affiliations:** Department of Pediatric Surgery, The Children's Hospital and the Institute of Child Health Lahore, Pakistan

A 45-day-old male infant presented with signs of acute intestinal obstruction for two days. Abdominal examination revealed a mass in the right lower abdomen. Ultrasound showed a cystic mass measuring 4x3 cm, in the right lower quadrant at the level of lower pole of right kidney. The plain abdominal radiograph showed haziness in the middle and lower third with bowel loops pushed to upper third of the abdomen (Fig. 1).

**Figure:**
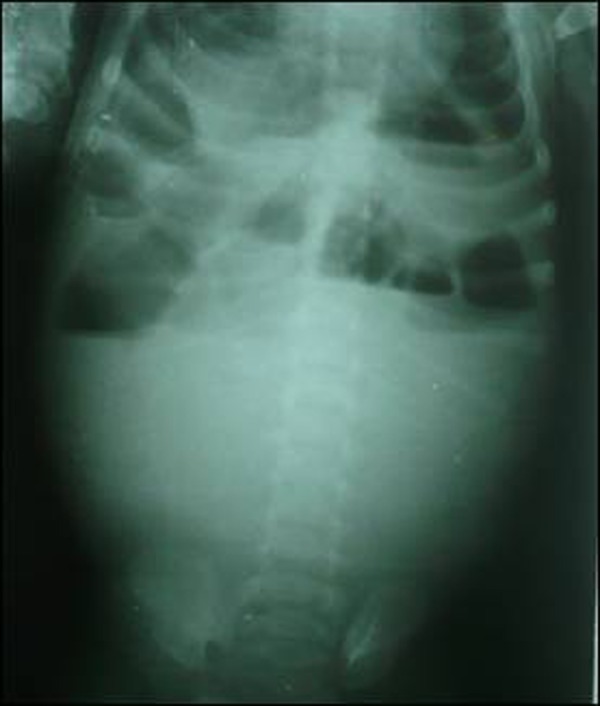
Figure 1: Abdominal radiograph suggestive of mass effect and intestinal obstruction

At operation, a cystic mass on the mesenteric side of the cecum was found. The small intestine was distended whereas large gut was collapsed 2). There was complete intestinal obstruction at the level of the cyst as appreciated by a failure of passage of the intestinal contents distally. The cystic mass was opened and about 50cc mucous drained. This resulted in sudden passage of intestinal contents into the ascending colon. The posterior wall of the cyst was being shared with the cecum. Mucosal stripping was performed after eversion. Patient made an uneventful recovery and discharged home on 4th post-operative day. Histopathology confirmed it as cecal duplication. 

**Figure:**
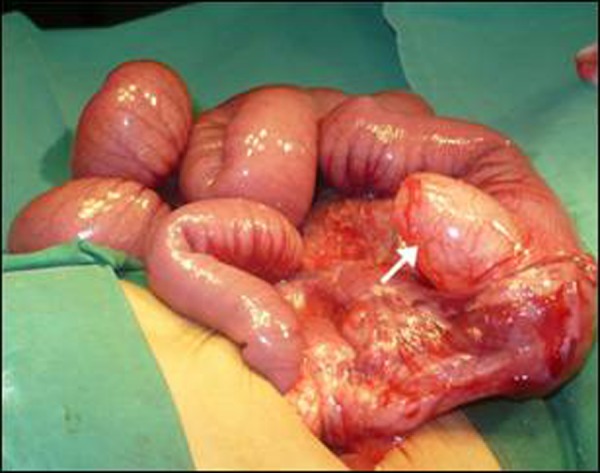
Figure 2: Operative view of the cecal duplication (arrow). The ascending colon was collapsed

## DISCUSSION

Duplications of the alimentary tract are rare anomalies. They may be of cystic or tubular variety with an intimate contact with the adjacent gut; smooth muscles in their wall and mucosa resemble that of intestine. Most common duplications occur along the ileum. Colonic duplications are rare (13%); cecal duplications are even rarer as only less than 20 cases have been reported in English literature [1, 2, 3].

They usually present within first two years of life in 80% of cases but has been reported in adults as well. Cecal duplications usually present with acute intestinal obstruction. They communicate with gut in less than 20% of cases where the presentation may be with bleeding per rectum if an ectopic gastric mucosa is present in it. Cecum is also a site where some colonic tubular duplications are intimately attached [1, 2, 3, 5, 6].

Ultrasound, CT scan, contrast bowel studies, technetium 99m radionuclide scan, and diagnostic laparoscopy are important tools for preoperative diagnosis; however, cecal duplications are diagnosed at operation in most of the cases. Our preoperative diagnosis was mesenteric cyst with a differential of alimentary tract duplication, not specifically of cecal origin.

The mechanism of obstruction in case of cecal duplication depends upon the amount of mucous in its lumen. Fully loaded cecal duplication can obstruct the lumen of the normal cecum and may result in acute intestinal obstruction as in our case. Simple drainage of the mucus relieved obstruction in the index case.

Various surgical procedures have been employed to deal with such lesions. Cecal duplications are commonly managed by limited right hemicolectomy and ileocolic anastomosis [1, 2, 3, 4, 5]. In our case drainage of the mucous relieved obstruction therefore anterior wall was completely excised and mucosal stripping was performed on the common wall. The common wall appeared thin and redundant thus plicated along with the margins of the residual anterior wall of the duplication. 

## Footnotes

**Source of Support:** Nil

**Conflict of Interest:** None declared
